# Treatment of idiopathic anaphylaxis with dupilumab: a case report

**DOI:** 10.1186/s13223-023-00838-8

**Published:** 2023-09-09

**Authors:** Elizabeth Pepper, Luke Pittman

**Affiliations:** 1 Department of Internal Medicine, Dwight D Eisenhower Army Medical Center, 300 E Hospital Street, Fort Gordon, GA 30905 USA; 2 Department of Allergy and Immunology, Dwight D Eisenhower Army Medical Center, 300 E Hospital Street, Fort Gordon, GA 30905 USA

**Keywords:** Idiopathic anaphylaxis, Dupilumab, Th2 inflammation, Omalizumab, Mast cell activation syndrome, Chronic spontaneous urticaria

## Abstract

**Background:**

Anaphylaxis is an acute, potentially life-threatening allergic reaction that typically occurs after exposure to a trigger, while idiopathic anaphylaxis (IA) occurs in the absence of a trigger. Acute management of both triggered anaphylaxis and IA relies on the use of epinephrine. In some patients with recurrent IA, glucocorticoid prophylaxis with prednisone can be effective. While there is currently no high quality evidence for the use of other prophylactic options to prevent recurrent IA, evolving data exists to support the consideration of biologics that target IgE or the Th2 pathway.

**Case presentation:**

We present the case of a 28 year old female with no atopic or autoimmune history with recurrent episodes of IA since childhood occurring up to twice weekly. There was improvement in acute symptoms with administration of first or second generation antihistamines and/or intramuscular epinephrine. Without an identifiable trigger, she was diagnosed with IA and frequent idiopathic urticaria and omalizumab was added to her treatment regimen with improvement in symptom frequency. After being lost to follow up, she had recurrence of symptom frequency and severity without omalizumab therapy and subsequently presented to our institution. Her workup at this point was negative for food allergy, alpha gal syndrome, systemic mastocytosis, hereditary alpha tryptasemia, carcinoid syndrome, and pheochromocytoma, and she was trialed on dupilumab with near resolution of her symptom frequency over a six month time period.

**Conclusion:**

Recurrent IA is a diagnosis of exclusion that is associated with high morbidity. Prophylaxis remains an area of uncertainty, although prednisone has been effective in some cases. When prednisone is contraindicated or ineffective for the prevention of IA, biologic therapies that target IgE or the Th2 pathway may present a reasonable consideration. This case adds support to the suggestion that dupilumab may be a logical off-label consideration for prophylaxis of recurrent IA. The data for dupilumab in this clinical scenario is still very limited, and further research is required before any recommendation can be made.

## Background

Anaphylaxis is an acute, potentially life-threatening allergic reaction that typically occurs after exposure to a trigger, while idiopathic anaphylaxis (IA) occurs in the absence of a trigger [1–3]. Acute management of both triggered anaphylaxis and IA relies on the use of epinephrine [1]. In some patients with recurrent IA, glucocorticoid prophylaxis with prednisone can be effective [2–4]. While there is currently no high quality evidence for the use of other prophylactic options to prevent recurrent IA, evolving data exists to support the consideration of biologics that target IgE or the Th2 pathway [5–9].

## Case Presentation

We present the case of a 28-year-old female with no atopic or autoimmune history who reported recurrent episodes of IA since childhood. These episodes occurred up to twice weekly and consisted of acute spontaneous pruritis, urticaria, flushing, diarrhea, tachycardia, lightheadedness, and presyncope or complete syncope without a consistent pattern or trigger. Her symptoms reportedly improved acutely with the use of first or second-generation antihistamines and/or intramuscular epinephrine. She reported previously failing chronic low-dose prednisone as a prophylactic agent without trialing high doses for prolonged periods of time. She was also not interested in a trial of higher dose prednisone in an attempt to provide tighter control of her symptoms due to poor tolerance of the medication. While she did report frequent emergency department visits for her episodes, she denied ever requiring intubation, hospitalization, or admission to the intensive care unit, with symptoms often resolving after self-treatment at home. Notably, she reported using an epinephrine autoinjector on an almost weekly basis and requested frequent EpiPen refill prescriptions. She reported being otherwise generally healthy, with a previous medical history consisting primarily of mental health concerns to include anxiety, panic disorder, and PTSD for which she received active care.

She was first evaluated by an allergist for symptomatic episodes in 2019 and found to have an elevated chronic urticaria index (13.1) and normal basal serum tryptase (6.6 mcg/L) with otherwise unremarkable basic labs. She was initiated on high dose antihistamines and given EpiPens to carry. She reported no significant improvement in episode frequency or severity and was subsequently placed on maximally dosed H1 and H2 blocking antihistamines and the leukotriene antagonist, montelukast. Despite this treatment strategy, she continued to report two to three episodes monthly. After one episode that she was unable to abort with self-treatment at home, she presented to the emergency room and was found to have an urticarial rash and an acutely elevated serum tryptase of 17.2 mcg/L with an otherwise unremarkable acute workup. She was treated medically and discharged home without the need for overnight observation.

She was diagnosed by her allergist with refractory IA and frequent idiopathic urticaria and started monthly 300 mg subcutaneous injections of omalizumab in August of 2020. She stated that omalizumab significantly improved her frequency of IA and chronic urticaria. Despite this, she continued to suffer frequent IA and eventually required increased frequency of omalizumab injections to 300 mg every three weeks to more adequately control her episodes. A note written by her allergist in November of 2020 stated, “[The patient] has self-administered EpiPen on at least 5 occasions over the last 3 months.”

Because of her concerning persistent symptomatology, she underwent bone marrow biopsy to assess for a clonal mast cell disorder. Her bone marrow was noted to be normocellular, devoid of mast cell aggregates or atypical mast cell populations, and negative for D816V KIT and BCR-ABL mutations. Of note, CD25 and/or tryptase staining were not performed and Sanger sequencing was used to assess for the D816V KIT mutation. The pathologist’s bone marrow biopsy report notes stated, “Normal numbers of singly scattered CD117 + mast cells…with no aggregates, atypical morphology, or aberrant CD2 expression.”

Despite the use of 300 mg subcutaneous omalizumab every three weeks, maximum dose antihistamines, and montelukast, she continued to suffer monthly IA episodes. When her allergist retired, she found it difficult to receive continued prescriptions for omalizumab via a local civilian allergist. She presented to the Eisenhower Army Medical Center Allergy Clinic in early 2022 and reported multiple episodes per month of IA requiring frequent use of EpiPens in the context of a lack of access to omalizumab for several months.

Extensive evaluation at our clinic included normal basic labs (including CBC, CMP, TSH, ESR, and CRP), negative digital droplet polymerase chain reaction D816V KIT mutation testing on peripheral blood, normal 24-hour urine N-methylhistamine, normal TPSAB1 copy number analysis, and undetectable serum specific IgE (sIgE) to alpha-gal and omega-5-gliadin. Her total IgE was 382 mg/dL, and serum immunoglobulins, 5-HIAA, vasoactive intestinal peptide, gastrin, calcitonin, and plasma metanephrines were all within normal range. Serum sIgE to a wide array of foods and aeroallergens revealed robust sensitization to tree pollens and a food sensitization profile consistent with in vitro cross-reactivity to tree pollen (monosensitization to Cor a1 and Ara h8 on hazelnut and peanut component panels in the setting of oral tolerance to all tested foods). Notably, repeat chronic urticaria index was again elevated at 39.7.

Given her report of monthly IA episodes even when previously on omalizumab at a frequency of every three weeks, our clinic started the patient on 300 mg subcutaneous dupilumab off-label injected every two weeks in October of 2022. After dupilumab initiation, she reported one spontaneous episode of urticaria and gastrointestinal symptoms in November of 2022 but subsequently noted complete resolution of urticaria and IA. As of the time of this publication, she has been symptom free with zero IA or urticaria episodes for at least six months while on dupilumab.

## Discussion

IA is a diagnosis of exclusion [4]. Our patient’s recurrent spontaneous episodes of subjective symptoms in conjunction with objective findings of mast cell mediator release as documented through Emergency Department visits (e.g. acutely elevated tryptase, urticaria) in the absence of another diagnosis are most consistent with IA. She underwent extensive evaluation to rule out other possible causes of her symptoms to include food allergy, alpha gal syndrome, systemic mastocytosis, hereditary alpha tryptasemia, carcinoid syndrome, and pheochromocytoma. Bone marrow biopsy was completed and—although perhaps higher sensitivity could have been achieved with allele-specific PCR for the assessment of D816V KIT mutation and CD25/tryptase staining—there were no major or minor criteria satisfied for the diagnosis of systemic mastocytosis [10]. Repeat bone marrow biopsy was not considered necessary in the setting of a low REMA score [11].

Dupilumab was chosen as off-label prophylactic therapy for this patient’s recurrent IA for multiple reasons. First, she reported poor tolerance and poor efficacy with a trial of prednisone in the past. Second, she reported significant but incomplete benefit with omalizumab. Third, dupilumab’s mechanism of action suppresses Th2 pathways upstream compared to omalizumab and limits the production of serum IgE, a key component of the immunologic degranulation of mast cells during anaphylaxis [12]. Fourth, dupilumab may be effective in the prevention of chronic spontaneous urticaria (CSU) refractory to omalizumab, and our patient reported urticaria with nearly all symptomatic episodes [13]. Finally, limited evidence suggests that dupilumab may be effective at preventing recurrent anaphylaxis [6, 9].

There are potential limitations to this case report. Perhaps most notably, there was significant reliance on patient-reported subjective symptoms with only one objectively confirmed episode of mast cell mediator release (via acutely elevated serum tryptase collected during an episode). Given the physician documentation of urticaria during that same ED visit, there is strong evidence that at least this episode was due to mast cell mediator release. The more interesting question may be whether or not her reported symptoms could have represented severe/refractory CSU in the setting of uncontrolled or poorly controlled anxiety or panic disorder. Evidence for such a consideration includes her elevated chronic urticaria index and history of multiple behavioral health disorders. Omalizumab is FDA-approved as an effective treatment for CSU, and cases refractory to omalizumab have been successfully treated with dupilumab [13]. While it would perhaps be expected for high dose antihistamines to be at least somewhat effective in the prophylaxis of her symptoms if CSU were the sole diagnosis, it is conceivable that she had a severe case of CSU requiring a more aggressive preventative therapy such as omalizumab or dupilumab. Interestingly, there are reports of patients with CSU demonstrating elevated serum tryptase levels during active urticaria episodes [14]. An alternative diagnostic consideration would be mast cell activation syndrome (MCAS), though it is unclear if such a distinction would affect treatment in this patient’s case [15]. Of note, one would suspect that resolution of urticaria alone would not lead to complete resolution of all reported symptoms if her episodes were in large part due to poorly controlled anxiety. Given the aforementioned considerations, we believe the conglomerate of evidence remains most supportive of the diagnosis of IA.

## Conclusion

Recurrent IA is a diagnosis of exclusion that is associated with high morbidity. Prophylaxis remains an area of uncertainty, although prednisone has been effective in some cases. When prednisone is contraindicated or ineffective for the prevention of IA, biologic therapies that target IgE or the Th2 pathway may present a reasonable consideration. This case adds support to the suggestion that dupilumab may be a logical off-label consideration for prophylaxis of recurrent IA. The data for dupilumab in this clinical scenario is still very limited, and further research is required before any recommendation can be made.

## Data Availability

Data sharing is not applicable to this article as no datasets were generated or analyzed during the current study.

## Abbreviations

IA: Idiopathic anaphylaxis
sIgE: serum specific IgE
MCAS: Mast cell activation syndrome
CSU: Chronic spontaneous urticaria
CBC: Complete blood count
CMP: Complete metabolic panel
TSH: Thyroid stimulating hormone
ESR: Erythrocyte sedimentation rate
CRP: C-reactive protein, REMA
